# The Association Between Sleep Health and a History of Cataract Surgery in the United States Based on the National Health and Nutrition Examination Survey (NHANES) 2005–2008

**DOI:** 10.3390/healthcare13101136

**Published:** 2025-05-13

**Authors:** Chuanxi Wang, Ning Bao, Zhengxuan Jiang

**Affiliations:** The Second Clinical College, Anhui Medical University, Hefei 230601, China; 2345012102@stu.ahmu.edu.cn

**Keywords:** sleep time, sleep trouble, sleep pattern, cataract surgery, cross-sectional study

## Abstract

**Background:** The aim of this study was to assess the relationship between sleep-related variables (sleep duration, sleep trouble, and sleep disorder), comprehensive sleep patterns, and the reported history of cataract surgery in the U.S. population aged 20 years and older. **Methods:** We utilized data from the National Health and Nutrition Examination Survey (NHANES) 2005–2008 database. First, we analyzed the association between covariates and the reported history of cataract surgery using univariable Poisson regression. Subsequently, we constructed three models to evaluate the association between sleep-related variables and the reported history of cataract surgery using multivariable Poisson regression. Subgroup analyses were conducted to determine whether the association between sleep and the reported history of cataract surgery exhibited heterogeneity. Finally, we performed a sensitivity analysis to assess the stability of the results. **Results:** A total of 8591 participants were included in this study, among whom 774 had a history of cataract surgery. After adjusting for all covariates, participants experiencing sleep trouble had a higher prevalence of reported history of cataract surgery than participants without sleep trouble [PR = 1.40; 95%CI = (1.22, 1.62)]. Regarding combined sleep, participants with poor sleep patterns had a 36% higher prevalence of reported history of cataract surgery than those with healthy sleep patterns [PR = 1.36; 95%CI = (1.13, 1.64)]. The results of the sensitivity analysis indicate that the relationship between sleep patterns and the reported history of cataract surgery is robust. **Conclusions:** Sleep trouble and poor sleep patterns are positively linked to the high prevalence of a reported history of cataract surgery. Further research is needed to explore the underlying mechanisms.

## 1. Introduction

Cataracts are the primary reason for blindness in developing countries, impacting approximately 20 million people worldwide [1]. There are primarily characterized by the opacity of the crystalline lens [2]. Epidemiological studies have identified several risk factors for cataracts, including age, obesity, diabetes, hypertension, smoking, and sun exposure [3,4,5]. Among these, age is the most significant risk factor, and there is extensive research indicating that the prevalence of cataracts increases with age [6]. Cataract surgery remains the treatment of choice, with the annual cost of cataract surgery in the United States estimated at USD 3.4 billion [7]. The aging population will cause a rise in cataract surgery, increasing socio-economic and public health burdens [8].

Healthy sleep is essential for learning, memory, and brain development [9,10] and is integral to a healthy lifestyle. To achieve optimal health, the Sleep Research Organization recommends that adults obtain at least 7 h of sleep per night [11]. Insufficient sleep time and poor sleep quality can adversely affect cardiovascular, reproductive, and mental health [12]. A cohort study showed an increased risk of cataracts in patients with sleep apnea [13]. A cross-sectional study in Korea found that both long and short sleep duration were associated with a high prevalence of diabetic retinopathy [14]. Sleep is closely linked to eye health. However, the relationship between sleep and cataracts remains unclear. Elucidating this relationship may be beneficial for the prevention and treatment of cataracts.

In our study, we analyzed data from the National Health and Nutrition Examination Survey (NHANES) database for 2005–2008 to research the connection between sleep-related variables (sleep duration, sleep disorder, and sleep trouble) and integrated sleep behaviors (defined as ‘sleep patterns’) and a reported history of cataract surgery.

## 2. Methods

### 2.1. Data and Study Sample

**Figure 1:**
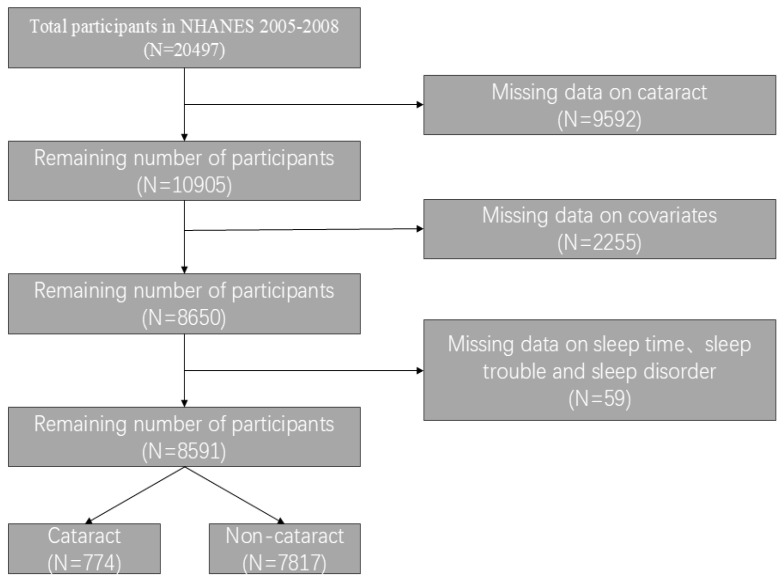
Flowchart of inclusion and exclusion criteria for the study population.

NHANES is a population-based cross-sectional survey conducted by the National Center for Health Statistics (NCHS) at the Centers for Disease Control and Prevention (CDC). Its purpose is to gather information on the health and nutrition of Americans in their households. This study included data from two NHANES cycles (2005–2006 and 2007–2008) with 20,497 participants. The exclusions for this study are as follows: (1) missing cataract surgery data; (2) missing self-reported data on sleep duration, sleep disorder, and sleep trouble; and (3) missing complete covariate data. Figure 1 illustrates the filtering process.

### 2.2. Cataract Surgery Assessment

The investigators assessed the history of cataract surgery by asking participants, “Have you ever had cataract surgery?” (VIQ071). If the answer was ‘YES’, the participant was diagnosed with a reported history of cataract surgery.

### 2.3. Assessment of Sleep-Related Variables

Falling asleep time was obtained by asking participants, “How long does it usually take you to fall asleep at bedtime?” The length of sleep was determined by asking participants, “How long do you usually sleep on weekdays or Sunday nights?”; we then divided the patients’ answers into short sleep periods (<7 h per night), healthy sleep periods (7–8 h per night), and long sleep periods (>8 h per night), in which short sleep and long sleep were both classed as unhealthy sleep. Sleep trouble and sleep disorders were assessed by the patient’s response to the questions ‘Have you ever told your doctor about your sleep problems?’ and ‘Has your doctor ever told you that you have a sleep disorder?’ The answers to these two questions were recorded. Participants’ sleep patterns were assessed using the three sleep behaviors described above. Healthy sleep duration, no sleep disorder, and no sleep trouble were scored with 1 point each. On the other hand, unhealthy sleep duration, sleep disorder, and sleep trouble were scored with 0, respectively. The scores for the three behaviors were summed to generate a composite sleep score. Detailed information is presented in Table S3. Based on the composite score, participants were categorized into three groups: healthy sleep pattern (3 points), intermediate sleep pattern (2 points), and poor sleep pattern (0–1 points) [15,16].

### 2.4. Assessment of Covariates

In line with previous studies on cataracts [17,18], we included covariates, including sociodemographic factors (sex, age, race, education level, marital status, and poverty–income ratio). Race was categorized as Mexican American, other Hispanic, non-Hispanic white, non-Hispanic black, and other race. Educational level was categorized as less than 9th grade, 9th-11th grade, high school graduate or equivalent, some college or AA degree, and college graduate or above. Economic level was assessed by the poverty–income ratio (PIR: household income/U.S. Department of Health and Human Services Federal Poverty Level). Based on the PIR, we classified the economic level into three categories (<1, 1–3, >3). A higher PIR indicates a better economic status of the household. Marital status was classified as married or living with a partner and unmarried or other. Sociodemographic factors, body measurements (body mass index (BMI)), lifestyle factors (smoking and alcohol consumption), and comorbidities (hypertension and diabetes) were obtained from self-reported questionnaires. The BMI was calculated by multiplying weight (kg) by height square (m^2^) and dividing it into three categories (<25, 25–30, >30 kg/m²). Two self-reported questionnaires assessed smoking and alcohol consumption. Participants were diagnosed with diabetes or hypertension if a doctor had told them that they had diabetes or hypertension.

### 2.5. Data Analysis

Given that NHANES uses complex multistage sampling and that we chose two consecutive cycles, 2-year moving examination center (MEC) weights divided by two were used to ensure that subjects were representative of the national population. On baseline descriptions, continuous variables are represented by means (SD) and categorized by frequency (percentage). The comparison of the baseline characteristics of the participants was performed using Wilcoxon rank tests and Rao–Scott chi-square tests. We then tested the association between the covariates of interest and the history of cataract surgery using univariable Poisson regression. To minimize confounding due to multicollinearity, the relationship between each sleep construct and the reported history of cataract surgery was tested individually in each one of Models 1, 2, and 3 for a total of 15 models: Model 1: unadjusted; Model 2: adjusted for sex, age and race; Model 3: adjusted for all covariates. Subgroup analyses were stratified by age, gender, race, educational level, marital status, economic level, BMI, alcohol consumption, smoking status, hypertension, and diabetes. Multiplicative interactions were assessed by including product terms between sleep patterns and the stratification variables in the models. Interaction *p*-values were obtained by comparing models with and without interaction terms using likelihood ratio tests. Finally, we use sensitivity analysis to test the reliability of the results. We analyzed the data using R4.3.2 (including “survey”, “dplyr”, and “foreign” packages), with a significant *p*-value < 0.05.

## 3. Results

### 3.1. Characteristics of Participants

Baseline characteristics were analyzed based on whether the subjects had undergone cataract surgery (Table 1). This study included 8591 subjects with complete information, of whom 774 had previously undergone cataract surgery. The subjects had an average age of 46.26 (16.55), with 51.8% identifying as women, primarily of a non-Hispanic white ethnicity.

Participants with poor sleep patterns had a mean age (standard deviation) of 49.75 (15.41) and were more likely to be female and less educated, have a lower economic level, be unmarried and obese, smoke and drink alcohol, and have hypertension and diabetes mellitus (Table 2). Additionally, a higher prevalence of a reported history of cataract surgery was associated with sleep patterns deteriorating.

### 3.2. Univariable Poisson Regression Analysis of Cataract-Related Variables

**Figure 2:**
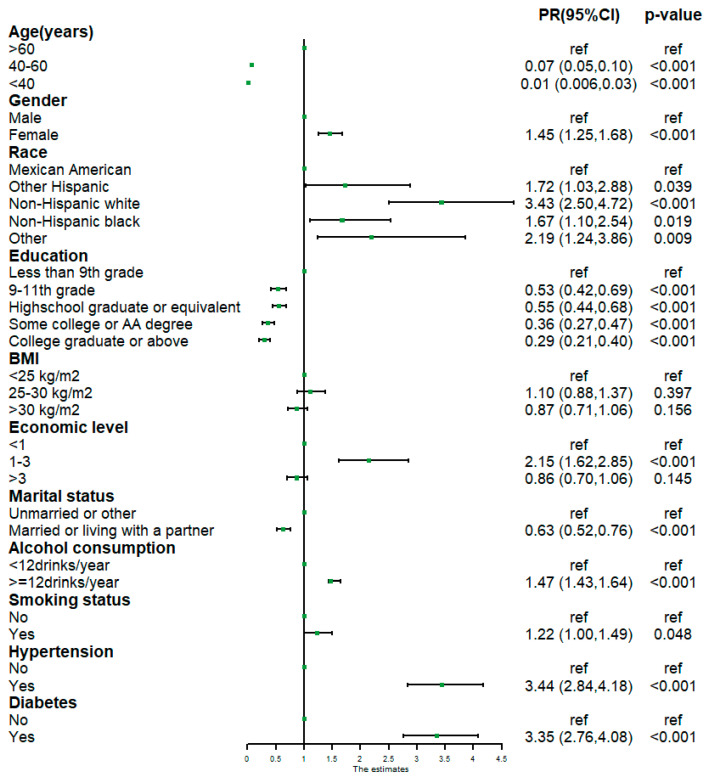
Univariable Poisson regression of the association between a history of cataract surgery and covariates. Abbreviations: PR: prevalence ratio; CI: confidence interval; BMI: body mass index.

Univariable Poisson regression was applied to analyze the association of covariables with a reported history of cataract surgery (Figure 2). We found that women had a 45% higher prevalence of a reported history of cataract surgery than men [PR = 1.45; 95%CI = 1.25, 1.68)]. Non-Hispanic whites had a higher prevalence of reported history of cataract surgery compared to Mexican Americans [PR = 3.43; 95%CI = (2.50, 4.72)]. Regarding education, a negative association was observed between participants’ education level and the prevalence of a reported history of cataract surgery. As for economic level, participants with an economic level between 1 and 3 had a 115% higher prevalence of a reported history of cataract surgery than those with an economic level < 1 [PR = 2.15; 95%CI = (1.62, 2.85)]. Regarding marital status, married participants had a lower prevalence of a reported history of cataract surgery than unmarried participants [PR = 0.63; 95%CI = (0.52, 0.76)]. Regarding lifestyle habits, the prevalence of a reported history of cataract surgery among smokers was higher compared to non-smokers [PR = 1.22; 95%CI = (1.00,1.49)]. Individuals who consumed more than 12 drinks per year had a higher prevalence of a reported history of cataract surgery than those who consumed less than 12 drinks per year [PR = 1.47; 95%CI = (1.43,1.64)]. Individuals with diabetes [PR = 3.35; 95%CI = (2.76,4.08)] or hypertension [PR = 3.44; 95%CI = (2.84,4.18)] had a higher prevalence of a reported history of cataract surgery than those without diabetes or hypertension.

### 3.3. Association Between Sleep and History of Cataract Surgery

Three multivariable Poisson regression models were employed to analyze the relationship between sleep and the prevalence of a reported history of cataract surgery (Table 3). In Model 3, participants with self-reported sleep trouble had a 40% higher prevalence of a reported history of cataract surgery compared to those without sleep trouble [PR = 1.40; 95%CI (1.22,1.62)]. In terms of sleep patterns, after adjusting for all covariates, participants with intermediate and poor sleep patterns showed 24% [PR = 1.24; 95% CI (1.05–1.46)] and 36% [OR = 1.36, 95% CI (1.13–1.64)] higher prevalence of reporting a history of cataract surgery, respectively, compared to those with healthy sleep patterns.

### 3.4. Subgroup Analyses

We conducted a subgroup analysis based on covariates to further explore the relationship between sleep trouble, sleep patterns, and a reported history of cataract surgery across different populations. The results (Table 4 and Table 5) showed no statistically significant differences between the subgroups, suggesting that factors such as sociodemographics, body measurements, lifestyle factors, and comorbidities did not significantly affect this association (all *p* for interaction > 0.05).

### 3.5. Sensitivity Analyses

We conducted three sensitivity analyses: 1. To account for the effects of extreme samples, we excluded participants who slept less than 3 hours (0.48%) and equal to 12 h (0.50%). After adjusting for all covariates, intermediate and poor sleep patterns participants had a higher prevalence of a reported history of cataract surgery compared to healthy sleep pattern participants [intermediate: PR (95%CI) = 1.24 (1.05,1.46); poor: PR (95%CI) = 1.36 (1.13,1.65)]. 2. Unweighted data were used. The results indicated that when the healthy sleep pattern was considered as a control, the PR (95% CI) for the intermediate and poor sleep patterns were 1.14 (0.96, 1.35) and 1.34 (1.09, 1.63), respectively. 3. We also adjusted for cardiovascular diseases, including congestive heart failure, coronary heart disease, angina, and stroke (N = 8521). The results of the multivariable Poisson regression showed that individuals with intermediate and poor sleep patterns had 24% and 39% higher prevalence of a reported history of cataract surgery compared to those with a healthy sleep pattern, respectively [intermediate: PR (95%CI) = 1.24 (1.03,1.49); poor: PR (95%CI) = 1.39(1.15,1.68)] (Tables S4–S6).

## 4. Discussion

This is the first study to utilize the NHANES data to explore the association between sleep patterns and the reported history of cataract surgery. Using multivariable Poisson regression analyses, we found that sleep trouble correlated with a reported history of cataract surgery. We then assessed the correlation between sleep patterns and a history of cataract surgery; participants with poor sleep patterns had a higher prevalence of underdoing cataract surgery. Sensitivity analyses further confirmed our findings.

Previous research has indicated that a short sleep duration is a significant risk factor for cataracts [19], while a long sleep duration has been found to have an insignificant association with cataracts [20]. We note that a Korean study showed that a longer sleep duration was linked to protection against cataract development [21]. In contrast, our findings show that there is no relationship between sleep duration and cataracts. The possible reasons for the conflicting results may be (1) the fact that different databases used, leading to differences in the study populations; (2) inconsistencies in the nadir criteria; (3) differences in the covariates adjusted for in the studies; and (4) the fact that some scientists have argued that a long sleep duration is not indicative of good sleep quality and it may be that poor sleepers increase their sleep by proxy [22,23].

In addition to sleep duration, participants who self-reported sleep problems had an increased risk of cataracts. This association may be related to some metabolic factors. Previous research has demonstrated that sleep deprivation is linked to hyperglycemia and poor blood sugar control [24,25] and disrupts circadian rhythms, affecting morning cortisol levels and sympathetic vagal homeostasis [26], which can lead to decreased insulin sensitivity [27] and an increased likelihood of diabetes [28]. Furthermore, numerous studies have demonstrated that sleep problems are linked to a higher chance of hypertension [29,30]. Diabetes and hypertension are known to be risk factors for cataracts. Chronic sleep disturbance can lead to metabolic imbalance, disrupting the intraocular environment and causing the degeneration of lens proteins, which may trigger the development of cataracts.

In recent years, researchers have increasingly recognized the importance of overall sleep quality. According to two studies, individuals who have poor overall sleep quality are at higher risk of cardiovascular disease and depression compared to those who have healthy sleep quality [31,32]. Consistently with their findings, our study found that participants with poor sleep patterns were more likely to have cataracts than those with healthy sleep patterns. This may be because unhealthy sleep patterns increase systemic oxidative stress and inflammation [33,34], which are critical pathological processes in cataract formation. Oxidative stress products impair the body’s antioxidant defense system, leading to the damage of lens proteins and the apoptosis of lens epithelial cells, which in turn contributes to the development of cataracts [35,36,37]. Secondly, poor sleep quality can disrupt normal cortisol rhythms [38], leading to the overactivation of the hypothalamic–pituitary–adrenal (HPA) axis and elevated serum cortisol levels. Increased cortisol levels may affect lens metabolism and accelerate the denaturation and aggregation of lens proteins, ultimately contributing to the development of cataracts [39,40].

We must acknowledge the limitations of this study. Firstly, because it was a cross-sectional study, we cannot establish a causal relationship between sleep and a history of cataract surgery. Secondly, obtaining sleep variables and assessing combined sleep patterns by self-report may result in recall bias and misclassification. Objective sleep testing tools are needed to assess this in the future. And more than 50% of participants were indeed excluded from the analysis due to missing data. However, this missingness was not random, and given the large overall sample size, we chose not to impute the missing values in order to preserve the integrity of the data. However, such an approach could introduce bias in the data. Thirdly, there was no adjustment for residuals and other confounding variables, which may have introduced bias. Fourth, since the study utilized public databases in the United States, the results may not accurately reflect the prevalence of cataract surgery in different regions. Finally, the timing of the cataract surgery is not stated in the history. And it is not known whether the sleep problem developed before or after the surgery.

## 5. Conclusions

Sleep trouble and poorer sleep patterns were associated with a higher prevalence of reporting a history of cataract surgery. This finding provides new ideas for the treatment and prevention of cataracts. However, the causal relationship between sleep and a reported history of cataract surgery must be investigated experimentally.

## Figures and Tables

**Table 1:** Characteristics of participants stratified by cataract surgery from NHANES 2005–2008.

	All	Non-Cataract Surgery	Cataract Surgery	*p*-Value
Number	8591	7817 (93.4)	774 (6.6)	
Gender (N, %)				<0.001
Male	4183 (48.2)	3819 (48.9)	364 (39.1)	
Female	4408 (51.8)	3998 (51.1)	410 (60.9)	
Age [years, mean (SD)]	46.26 (16.55)	44.43 (15.28)	72.22 (11.48)	<0.001
Ethnicity (N, %)				<0.001
Mexican American	1556 (7.9)	1497(8.2)	59 (2.7)	
Other Hispanic	602 (3.9)	562(4.0)	40 (2.3)	
Non-Hispanic white	4277 (72.1)	3730(71.2)	547 (84.8)	
Non-Hispanic black	1830 (10.9)	1721(11.2)	109 (6.2)	
Other	326 (5.3)	307(5.4)	19 (3.9)	
Education (N, %)				<0.001
Less than 9th grade	997 (5.9)	835 (5.4)	162 (13.2)	
9th–11th grade	1417 (12.2)	1290 (12.1)	127 (14.6)	
High school graduate or equivalent	2078 (24.8)	1874 (24.4)	204 (30.3)	
Some college or AA degree	2366 (30.6)	2200 (31.0)	166 (24.4)	
College graduate or above	1733 (26.5)	1618 (27.2)	115 (17.4)	
BMI (N, %)				0.085
<25 kg/m^2^	2554 (32.5)	2318 (32.4)	236 (32.8)	
25–30 kg/m^2^	2981 (33.9)	2689 (33.6)	292 (37.6)	
>30 kg/m^2^	3056 (33.7)	2810 (34.0)	246 (29.6)	
Economic level (N, %)				<0.001
<1	1596 (12.0)	1481 (12.3)	115 (9.0)	
1–3	3640 (35.8)	3189 (34.3)	451 (57.6)	
>3	3355 (52.1)	3147 (53.4)	208 (33.4)	
Marital status (N, %)				<0.001
Unmarried or other	5350 (65.6)	4941 (66.4)	409 (54.5)	
Married or living with a partner	3241 (34.4)	2876 (33.6)	365 (45.5)	
Alcohol consumption (N, %)				<0.001
≥12 drinks/year	6028 (74.8)	5567 (75.8)	461 (61.1)	
<12 cups/year	2563 (25.2)	2250 (24.2)	313 (38.9)	
Smoking status (N, %)				0.047
Yes	4115 (48.2)	3700 (47.8)	415 (53.2)	
No	4476 (51.8)	4117 (52.2)	359 (46.8)	
Hypertension (N, %)				<0.001
Yes	2876 (29.8)	2407 (27.7)	469 (59.4)	
No	5715 (70.2)	5410 (72.3)	305 (40.6)	
Diabetes (N, %)				<0.001
Yes	963 (8.0)	767 (6.9)	196 (22.5)	
No	7628 (92.0)	7050 (93.1)	578 (77.5)	
Sleep time (N, %)				<0.001
<7	3330 (36.4)	3060 (36.6)	270 (33.4)	
7–9	5030 (61.3)	4570 (61.4)	460 (61.1)	
>9	231 (2.2)	187 (2.0)	44 (5.4)	
Falling asleep time [min, mean (SD)]	21.36 (19.12)	21.21 (19.03)	23.42 (20.21)	0.050
Sleep trouble (N, %)				<0.001
Yes	1945 (24.3)	1698 (23.5)	247 (34.6)	
No	6646 (75.7)	6119 (76.5)	527 (65.4)	
Sleep disorder (N, %)				0.515
Yes	619 (7.3)	550 (7.3)	69 (7.9)	
No	7972 (92.7)	7267 (92.7)	705 (92.1)	
Sleep patterns (N, %)				<0.001
Healthy sleep	4121 (48.7)	3780 (49.0)	341 (43.9)	
Intermediate sleep	3115 (35.5)	2846 (35.6)	269 (34.5)	
Poor sleep	1355 (15.8)	1191 (15.4)	164 (21.6)	

Abbreviations: SD: standard deviation; BMI: body mass index.

**Table 2:** Characteristics of participants stratified by sleep pattern from NHANES 2005–2008.

	Sleep Pattern (Col%)			
	Healthy	Intermediate	Poor	*p*-Value
Number	3791 (48.7)	3387 (35.5)	1413 (15.8)	
Gender (N, %)				0.003
Male	1888 (49.6)	1685 (49.5)	610 (41.8)	
Female	1903 (50.4)	1702 (50.5)	803 (58.2)	
Age [years, mean (SD)]	45.47 (16.70)	45.66 (16.67)	49.75 (15.41)	<0.001
Ethnicity (N, %)				<0.001
Mexican American	832 (9.5)	574 (7.5)	150 (4.2)	
Other Hispanic	285 (4.3)	214 (3.7)	103 (3.4)	
Non-Hispanic White	1917 (72.8)	1569 (69.2)	791 (76.7)	
Non-Hispanic Black	614 (8.0)	898 (14.2)	318 (11.1)	
Other	143 (5.4)	132 (5.5)	51 (4.6)	
Education (N, %)				<0.001
Less than 9th grade	482 (6.3)	356 (5.3)	159 (6.0)	
9th–11th grade	614 (11.5)	588 (13.3)	215 (11.8)	
High school graduate or equivalent	868 (22.9)	842 (25.7)	368 (27.8)	
Some college or AA degree	982 (28.7)	952 (31.3)	432 (33.9)	
College graduate or above	845 (30.6)	649 (24.3)	239 (20.4)	
BMI (N, %)				<0.001
<25 kg/m ^2^	1229 (35.9)	996 (31.3)	329 (25.7)	
25–30 kg/m ^2^	1392 (34.7)	1171 (35.1)	418 (28.6)	
>30 kg/m ^2^	1170 (29.4)	1220 (33.6)	666 (45.6)	
Economic level (N, %)				0.001
<1	682 (11.2)	593 (11.4)	321 (15.9)	
1–3	1556 (33.5)	1500 (38.9)	584 (35.1)	
>3	1553 (55.3)	1294 (49.7)	508 (49.0)	
Marital status (N, %)				<0.001
Unmarried or other	1278 (31.1)	1340 (35.8)	614 (39.9)	
Married or living with a partner	2504 (68.9)	2047 (64.2)	799 (60.1)	
Alcohol consumption (N, %)				0.009
≥12 drinks/year	2684 (75.6)	2385 (75.5)	959 (71.3)	
<12 cups/year	1107 (24.4)	1002 (24.5)	454 (28.7)	
Smoking status (N, %)				<0.001
Yes	1692 (44.7)	1612 (48.6)	811 (56.6)	
No	2099 (55.3)	1775 (51.4)	602 (43.4)	
Hypertension (N, %)				<0.001
Yes	1024 (24.3)	1119 (29.7)	732 (44.9)	
No	2767 (75.7)	2268 (70.3)	680 (55.1)	
Diabetes (N, %)				<0.001
Yes	340 (6.3)	356 (7.0)	267 (14.8)	
No	3451 (93.7)	3031 (93.0)	1146 (85.1)	
Cataract surgery (N, %)				
Yes	289 (5.3)	310 (6.9)	175 (9.1)	<0.001
No	3502 (94.7)	3077 (93.1)	1238 (90.9)	

Abbreviations: SD: standard deviation; BMI: body mass index.

**Table 3:** The correlation between sleep and a history of cataract surgery.

Variable	Model 1		Model 2		Model 3	
	PR (95%CI)	*p*-Value	PR (95%CI)	*p*-Value	PR (95%CI)	*p*-Value
**Sleep duration (continuous)**	1.15 (1.07, 1.24)	**<0.001**	0.99 (0.94, 1.05)	0.834	1.00 (0.94, 1.06)	0.996
**Sleep duration (multi-category)**						
7–8 h	ref		ref	ref	ref	
<7 h	1.00 (0.78, 1.27)	0.97	1.12 (0.91, 1.38)	0.284	1.07 (0.87, 1.32)	0.463
>8 h	2.26 (1.77, 2.90)	**<0.001**	1.21 (0.97, 1.51)	0.083	1.17 (0.91, 1.49)	0.196
**Sleep disorder**						
No	ref		ref		ref	
Yes	1.10 (0.83, 1.45)	0.514	1.16 (0.88, 1.53)	0.273	1.11 (0.82, 1.50)	0.455
**Sleep trouble**						
No	ref		ref		ref	
Yes	1.65 (1.46, 1.86)	**<0.001**	1.46 (1.29, 1.66)	**<0.001**	1.40 (1.22, 1.62)	**<0.001**
**Sleep patterns**						
Healthy sleep	ref		ref		ref	
Intermediate sleep	1.29 (1.06, 1.57)	**0.0117**	1.27 (1.09, 1.49)	**0.004**	1.24 (1.05, 1.46)	**0.017**
Poor sleep	1.70 (1.38, 2.10)	**<0.001**	1.46 (1.23, 1.74)	**<0.001**	1.36 (1.13, 1.64)	**0.004**

Model 1: unadjusted. Model 2: Model 1 + sex, age, and race. Model 3: Model 2 + educational level, marital status, economic level, BMI, alcohol consumption, smoking status, hypertension, and diabetes. Abbreviations: BMI: body mass index; PR: prevalence ratio; CI: confidence interval.

**Table 4:** Subgroup analyses of the association between sleep patterns and cataract surgery.

		Healthy Sleep	Intermediate Sleep		Poor Sleep		*p* for Interaction
Subgroup			PR (95%CI)	*p*-Value	PR (95%CI)	*p*-Value	
**Age (years)**							0.249
<60	51/5796	ref	2.13 (0.91, 4.99)	0.076	2.46 (0.95, 6.35)	0.061	
≥60	723/2795	ref	1.18 (0.95, 1.47)	0.116	1.29 (1.04, 1.61)	0.025	
**Gender**							0.867
Male	364/4183	ref	1.19 (0.85, 1.66)	0.291	1.39 (0.93, 2.08)	0.102	
Female	410/4408	ref	1.27 (1.06, 1.53)	0.015	1.37 (1.12, 1.69)	0.006	
**Ethnicity**							0.832
Mexican American	59/1556	ref	0.90 (0.53, 1.52)	0.660	1.55 (1.02, 2.34)	0.040	
Other Hispanic	40/602	ref	1.60 (0.60, 4.27)	0.270	1.57 (0.25, 9.95)	0.561	
Non-Hispanic white	547/4277	ref	1.26 (1.06, 1.49)	0.011	1.39 (1.14, 1.70)	0.003	
Non-Hispanic black	109/1830	ref	1.15 (0.82, 1.62)	0.389	1.28 (0.88, 1.87)	0.185	
Other	19/326	ref	0.92 (0.23, 3.73)	0.896	1.21 (0.25, 5.72)	0.798	
**Education**							0.939
Less than 9th grade	162/997	ref	1.16 (0.74, 1.82)	0.486	1.05 (0.75, 1.47)	0.761	
9th–11th grade	127/1417	ref	1.41 (0.92, 2.17)	0.109	1.71 (1.03, 2.84)	0.040	
High school graduate or equivalent	204/2078	ref	1.29 (0.99, 1.68)	0.062	1.55 (1.04, 2.31)	0.033	
Some college or AA degree	166/2366	ref	1.15 (0.83, 1.60)	0.379	1.31 (0.79, 2.18)	0.275	
College graduate or above	155/1773	ref	1.30 (0.79, 2.15)	0.281	1.37 (0.69, 2.71)	0.338	
**BMI**							0.062
<25 kg/m^2^	236/2554	ref	1.36 (1.05, 1.75)	0.023	1.32 (0.91, 1.93)	0.133	
25–30 kg/m^2^	292/2981	ref	1.37 (0.99, 1.89)	0.058	1.13 (0.82, 1.55)	0.432	
>30 kg/m^2^	246/3056	ref	0.79 (0.54, 1.16)	0.208	1.48 (1.02, 2.41)	0.040	
**Economic level**							0.465
<1	115/1596	ref	0.86 (0.48, 1.53)	0.576	1.33 (0.68, 2.64)	0.374	
1–3	451/3640	ref	1.21 (1.00, 1.47)	0.052	1.32 (1.02, 1.71)	0.040	
>3	208/3355	ref	1.45 (1.03, 2.04)	0.034	1.41 (0.90, 2.21)	0.119	
**Marital status**							0.562
Unmarried or other	365/3241	ref	1.10 (0.88, 1.39)	0.361	1.21 (0.89, 1.66)	0.198	
Married or living with a partner	409/5350	ref	1.33 (1.08, 1.64)	0.011	1.44 (1.02, 2.04)	0.040	
**Alcohol consumption**							0.751
≥12 drinks/year	461/6028	ref	1.17 (0.94, 1.44)	0.142	1.31 (1.02, 1.69)	0.034	
<12 cups/year	313/2563	ref	1.33 (1.00, 1.76)	0.047	1.43 (1.04, 1.97)	0.032	
**Smoking status**							0.551
Yes	415/4115	ref	1.20 (0.91, 1.56)	0.173	1.45 (1.13, 1.87)	0.007	
No	359/4476	ref	1.26 (1.04, 1.54)	0.024	1.28 (0.93, 1.72)	0.127	
**Hypertension**							0.785
Yes	469/2876	ref	1.24 (1.01, 1.52)	0.044	1.29 (0.97, 1.70)	0.072	
No	305/5715	ref	1.20 (0.90, 1.60)	0.188	1.46 (0.96, 2.22)	0.075	
**Diabetes**							0.965
Yes	196/963	ref	1.18 (0.86, 1.60)	0.271	1.32 (0.91, 1.91)	0.123	
No	578/7628	ref	1.24 (1.01, 1.52)	0.043	1.36 (1.06, 1.73)	0.020	

Abbreviations: BMI: body mass index; PR: prevalence ratio; CI: confidence interval. Each stratification is adjusted for all covariates.

**Table 5:** Subgroup analyses of the association between sleep trouble and a history of cataract.

Subgroup		PR (95%CI)	*p*-Value	*p* for Interaction
**Age (years)**				0.338
<60	51/5796	1.84 (1.00, 3.41)	0.049	
≥60	723/2795	1.30 (1.12, 1.51)	0.002	
**Gender**				0.448
Male	364/4183	1.29 (0.98, 1.70)	0.070	
Female	410/4408	1.50 (1.29, 1.74)	<0.001	
**Ethnicity**				0.617
Mexican American	59/1556	2.37 (1.39, 4.03)	0.004	
Other Hispanic	40/602	1.41 (0.36, 5.54)	0.564	
Non-Hispanic white	547/4277	1.43 (1.23, 1.66)	<0.001	
Non-Hispanic black	109/1830	1.02 (0.67, 1.47)	0.969	
Other	19/326	2.63 (0.52, 13.19)	0.218	
**Education**				0.611
Less than 9th grade	162/997	1.16 (0.86, 1.56)	0.321	
9th–11th grade	127/1417	1.63 (1.14, 2.33)	0.010	
High school graduate or equivalent	204/2078	1.59 (1.16, 2.18)	0.006	
Some college or AA degree	166/2366	1.55 (1.06, 2.27)	0.027	
College graduate or above	155/1773	1.22 (0.79, 1.88)	0.350	
**BMI**				0.495
<25 kg/m ^2^	236/2554	1.26 (0.97, 1.63)	0.075	
25–30 kg/m ^2^	292/2981	1.36 (1.02, 1.81)	0.040	
>30 kg/m ^2^	246/3056	1.63 (1.19, 2.24)	0.005	
**Economic level**				0.948
<1	115/1596	1.32 (0.81, 2.14)	0.238	
1–3	451/3640	1.38 (1.13, 1.68)	0.004	
>3	208/3355	1.44 (1.10, 1.89)	0.012	
**Marital status**				0.901
Unmarried or other	409/5350	1.43 (1.08, 1.87)	0.015	
Married or living with a partner	365/3241	1.33 (1.05, 1.67)	0.020	
**Alcohol consumption**				0.319
≥12 drinks/year	461/6028	1.34 (1.11, 1.61)	0.005	
<12 cups/year	313/2563	1.53 (1.20, 1.96)	0.002	
**Smoking status**				0.443
Yes	415/4115	1.35 (1.10, 1.66)	0.008	
No	359/4476	1.49 (1.18, 1.89)	0.003	
**Hypertension**				0.599
Yes	469/2876	1.34 (1.13, 1.58)	0.002	
No	305/5715	1.50 (1.11, 2.03)	0.013	
**Diabetes**				0.433
Yes	196/963	1.54 (1.14, 2.07)	0.008	
No	578/7628	1.36 (1.15, 1.60)	0.002	

Abbreviations: BMI: body mass index; PR: prevalence ratio; CI: confidence interval. Each stratification is adjusted for all covariates.

## Data Availability

Data available on request due to restrictions. NHANES is a public database and all researchers have access to the data from www.cdc.gov/nchs/nhanes, accessed on 15 March 2025.

## Supplementary Materials

The following supporting information can be downloaded at: https://www.mdpi.com/article/10.3390/healthcare13101136/s1, Table S1: The unadjusted rates of history of cataract surgery and no history of cataract surgery in different subgroups; Table S2: The unadjusted rates of different sleep pattern in different subgroups; Table S3: The overall sleep quality according to each sleep behavior; Table S4: The relationship between sleep and cataract surgery after excluding extreme values; Table S5: The relationship between sleep and cataract surgery after using unweighted data; Table S6: The relationship between sleep and cataract surgery after including cardiovascular diseases.
