# Supplementary material for: The Association Between Sleep Health and a History of Cataract Surgery in the United States Based on the National Health and Nutrition Examination Survey (NHANES) 2005–2008
Source: Healthcare (Basel). 2025 May 13;13(10):1136. doi: 10.3390/healthcare13101136 (PMC12111724; doi:10.3390/healthcare13101136)
Supplement: Supplementary file 1 [file healthcare-13-01136-s001.zip › healthcare-3566703-supplementary.pdf]

Supplementary Materials:

Table S1. The unadjusted rates of history of cataract surgery and no history of cataract surgery in different subgroups.

	All	Non-cataract surgery	Cataract surgery
Number	8591	7817(93.4)	774(6.6)
Gender (N, %)			
Male	4183	3819(91.3)	364(8.7)
Female	4408	3998 (90.7)	410 (9.3)
Age [years, mean (SD)]	46.26	44.43 (15.28)	72.22 (11.48)
Ethnicity (N, %)			
Mexican American	1556	1497(96.2)	59 (3.8)
Other Hispanic	602	562(93.4)	40 (6.6)
Non-Hispanic White	4277	3730(87.2)	547 (12.8)
Non-Hispanic Black	1830	1721(94.0)	109 (6.0)
Other	326	307(94.2)	19 (5.8)
Education (N, %)			
Less Than 9th Grade	997	835 (83.8)	162 (16.2)
9-11th Grade	1417	1290 (91.0)	127 (9.0)
Highschool graduate or equivalent	2078	1874 (90.2)	204 (9.8)
Some College or AA degree	2366	2200 (93.0)	166 (7.0)
College graduate or above	1733	1618 (93.4)	115 (6.6)
BMI (N, %)			
<25 kg/m ^{2}	2554	2318 (90.8)	236 (9.2)
25-30 kg/m ^{2}	2981	2689 (90.2)	292 (9.8)
>30 kg/m ^{2}	3056	2810 (92.0)	246 (8.0)
Economic level (N, %)			
<1	1596	1481 (92.8)	115 (7.2)
1-3	3640	3189 (87.5)	451 (12.5)
>3	3355	3147 (93.8)	208 (6.2)
Marital status (N, %)			
Unmarried or other	5350	4941 (92.4)	409 (7.6)
Married or living with a partner	3241	2876 (88.7)	365 (11.3)
Alcohol consumption (N, %)			
≥12 drinks/year	6028	5567 (92.4)	461 (7.6)
<12 cups/year	2563	2250 (87.8)	313 (12.2)
Smoking status (N, %)			
Yes	4115	3700 (89.9)	415 (10.1)
No	4476	4117 (92.0)	359 (8.0)
Hypertension (N, %)			
Yes	2876	2407 (83.7)	469 (16.3)
No	5715	5410 (94.7)	305 (5.3)
Diabetes (N, %)			
Yes	963	767 (79.6)	196 (20.1)
No	7628	7050 (92.4)	578 (7.6)
Sleep time (N, %)			
<7	3330	3060 (91.9)	270 (8.1)
7-9	5030	4570 (90.9)	460 (9.1)
>9	231	187 (81.0)	44 (19.0)
Fall time [min, mean (SD)]	21.36 (19.12)	21.21 (19.03)	23.42 (20.21)

Sleep trouble (N, %)			
Yes	1945	1698 (87.3)	247 (12.7)
No	6646	6119 (92.1)	527 (7.9)
Sleep disorder (N, %)			
Yes	619	550 (88.9)	69 (11.1)
No	7972	7267 (91.2)	705 (8.8)
Sleep patterns (N, %)			
Healthy sleep	3791	3502 (92.4)	289 (7.6)
Intermediate sleep	3387	3077 (90.8)	310 (9.2)
Poor sleep	1413	1238 (87.6)	175 (12.4)

Abbreviations: SD: Standard Deviation; BMI: body mass index.

Table S2. The unadjusted rates of different sleep pattern in different subgroups.

	Sleep pattern (Col%)		
	**Healthy**	**Intermediate**	Poor
Number	3791(44.1)	3387(39.4)	1413(16.4)
Gender (N, %)			
Male	1888 (45.1)	1685 (40.3)	610 (14.6)
Female	1903 (43.2)	1702 (38.6)	803 (18.2)
Age [years, mean (SD)]	45.47 (16.70)	45.66 (16.67)	49.75 (15.41)
Ethnicity (N, %)			
Mexican American	832 (53.5)	574 (36.9)	150 (9.6)
Other Hispanic	285 (47.3)	214 (35.5)	103 (17.1)
Non-Hispanic White	1917 (44.8)	1569 (36.7)	791 (18.5)
Non-Hispanic Black	614 (33.6)	898 (49.1)	318 (17.4)
Other	143 (43.9)	132 (40.5)	51 (15.6)
Education (N, %)			
Less Than 9th Grade	482 (48.3)	356 (35.7)	159 (15.9)
9-11th Grade	614 (43.3)	588 (41.5)	215 (15.2)
Highschool graduate or equivalent	868 (41.8)	842 (40.5)	368 (17.7)
Some College or AA degree	982 (41.5)	952 (40.2)	432 (18.3)
College graduate or above	845 (47.7)	649 (36.6)	239 (13.5)
BMI (N, %)			
<25 kg/m ^{2}	1229 (48.1)	996 (39.0)	329 (12.9)
25-30 kg/m ^{2}	1392 (46.7)	1171 (39.3)	418 (14.0)
>30 kg/m ^{2}	1170 (38.3)	1220 (39.9)	666 (21.8)
Economic level (N, %)			
<1	682 (42.7)	593 (37.2)	321 (20.1)
1-3	1556 (42.7)	1500 (41.2)	584 (16.0)
>3	1553 (46.3)	1294 (38.6)	508 (15.1)
Marital status (N, %)			
Unmarried or other	1287 (39.7)	1340 (41.3)	614 (18.9)
Married or living with a partner	2504 (46.8)	2047 (38.3)	799 (13.9)
Alcohol consumption (N, %)			
≥12 drinks/year	2684 (44.5)	2385 (39.6)	959 (15.9)
<12 cups/year	1107 (43.2)	1002 (39.1)	454 (17.7)
Smoking status (N, %)			
Yes	1692 (41.1)	1612 (39.2)	811 (19.7)

No	2099 (46.9)	1775 (39.7)	602 (13.4）
Hypertension (N, %)			
Yes	1024 (35.6)	1119 (38.9)	732 (25.5)
No	2767 (48.4)	2268 (39.7)	680 (11.9)
Diabetes (N, %)			
Yes	340 (35.3)	356 (37.0)	267 (27.7)
No	3451 (45.2)	3031 (39.7)	1146 (15.0)
Cataract surgery (N, %)			
Yes	289 (37.3)	310 (40.1)	175 (22.6)
No	3502 (44.8)	3077 (39.4)	1238 (15.8)

Abbreviations: SD: Standard Deviation; BMI: body mass index.

Table S3. The overall sleep quality according to each sleep behavior

**Sleep parameters**	**Score**
Sleep duration	
<7h	**0**
7-8h	**1**
>8h	**0**
Sleep trouble	
Yes	**0**
No	**1**
Sleep disorder	
Yes	**0**
no	**1**
Total score	**0(worst)-3(best)**

Table S4. The relationship between sleep and reported history of cataract surgery after excluding extreme values.

	**PR (95%CI)**	***P*-value**
**Sleep duration (continuous)**	1.01 (0.94,1.07)	0.961
**Sleep duration (multi-category)**		
7-8h	ref	
<7h	1.08 (0.87,1.33)	0.462
>8h	1.18 (0.92,1.52)	0.174
**Sleep disorder**		
No	ref	
Yes	1.12 (0.84,1.51)	0.406
**Sleep trouble**		
No	ref	
Yes	1.41 (1.22,1.63)	**<0.001**
**Sleep patterns**		
Healthy sleep	ref	
Intermediate sleep	1.24 (1.05,1.46)	**0.015**
Poor sleep	1.36 (1.13,1.65)	**0.004**

Adjusted for age, gender, race, educational level, marital status, economic level, BMI, alcohol consumption, smoking status, hypertension, and diabetes. Abbreviations: BMI: body mass index; PR: Prevalence Ratio; CI: confidence interval.

Table S5. The relationship between sleep and reported history of cataract surgery after using unweighted data.

Variable	Model 1		Model 2		Model 3	
	PR (95%CI)	*p*-value	PR (95%CI)	*p*-value	PR (95%CI)	*p*-value
**Sleep time (continuous)**	1.12 (1.07,1.17)	<0.001	0.99 (0.95, 1.04)	0.780	1.00 (0.95, 1.05)	0.897
**Sleep time (multi-category)**						
7-8h	ref		ref		ref	
<7h	0.95 (0.82, 1.10)	0.508	1.07 (0.91, 1.26)	0.421	1.04 (0.88, 1.23)	0.630
>8h	2.02 (1.65, 2.47)	<0.001	1.19 (0.95, 1.48)	0.124	1.15 (0.92, 1.43)	0.216
**Sleep disorder**						
No	ref		ref		ref	
Yes	1.26 (0.99, 1.58)	0.054	1.31 (1.00, 1.69)	0.044	1.21 (0.92, 1.57)	0.159
**Sleep trouble**						
No	ref		ref		ref	
Yes	1.60 (1.38,1.85)	<0.001	1.43 (1.21, 1.68)	<0.001	1.36 (1.15, 1.60)	**<0.001**
**Sleep patterns**						
Healthy sleep	ref		ref		ref	
Intermediate sleep	1.20 (1.03, 1.40)	0.019	1.17 (0.99, 1.39)	0.073	1.14 (0.96, 1.35)	**0.142**
Poor sleep	1.62 (1.36, 1.94)	<0.001	1.44 (1.17, 1.75)	<0.001	1.34 (1.09, 1.63)	**0.005**

Model 1: unadjusted. Model 2: Model 1 + sex, age and race. Model 3: Model 2 + educational level, marital status, economic level, BMI, alcohol consumption, smoking status, hypertension, and diabetes. Abbreviations: BMI: body mass index; PR: Prevalence Ratio; CI: confidence interval.

Table S6. The relationship between sleep and reported history of cataract surgery after including cardiovascular diseases.

	PR (95%CI)	*P*-value
**Sleep duration (continuous)**	1.00(0.94,1.06)	0.926
**Sleep duration (multi-category)**		
7-8h	ref	
<7h	1.07(0.86,1.33)	0.476
>8h	1.18(0.90,1.55)	0.197
**Sleep disorder**		
No	ref	
Yes	1.13(0.83,1.54)	0.384
**Sleep trouble**		
No	ref	
Yes	1.43(1.23,1.67)	**<0.001**
**Sleep patterns**		
Healthy sleep	ref	
Intermediate sleep	1.24(1.03,1.49)	**0.027**
Poor sleep	1.39(1.15,1.68)	**0.005**

Adjusted for age, gender, race, educational level, marital status, economic level, BMI, alcohol consumption, smoking status, hypertension, diabetes, congestive heart failure, coronary heart disease, angina and stroke. Abbreviations: BMI: body mass index; PR: Prevalence Ratio; CI: confidence interval.
